# The Medical Profession and the Insurance Bill

**Published:** 1911-07-08

**Authors:** S. W. Daw

**Affiliations:** Resident Surgical Officer Leeds General Infirmary. General Infirmary, Leeds.


### THE EDITOR'S LETTER-BOX.

### THE MEDICAL PROFESSION AND THE INSURANCE BILL.

*To the Editor of* The Hospital.

Sir,—I wish to enter a protest against the attitude adopted in your article on "The Medical Profession and the Bill" in to-day's issue, page 332. You suggest that the impression has been given that the objections of medical men are primarily founded on self-interest. I would ask what is wrong with such an impression, and why should medical men be regarded as other than human? If a man's house should catch fire and he does his best to put the fire out, no reasonable person condemns his attitude as being one prompted by self-interest. Of course it is such, and rightly so.

The Insurance Bill, as regards doctors, is one of two things, either a demand for compulsory charity on the part of the doctors or a business proposition. If the former, the doctor has every right to object to being forced to be charitable; if the latter, he has every right to discuss or refuse the business proposition, and no one ought to do other than applaud him in either case. Also I cannot agree with your joining the Chancellor in speaking as if the doctors were trying to grab something and making "demands." All that is wanted by medical men is that they should be let alone to carry on what may fairly be considered a legitimate occupation, or that if something is demanded from the doctors they should have an adequate return, which is a very different matter from grabbing on their part. I think that on further consideration you must agree that it is hardly right to close the discussion on the medical aspect of the case without an expression of greater sympathy on your part for the legitimiate upholding of their rights by medical men. I am not in general practice, but I am quite in accord with general practitioners in looking after their own interests.—I am, yours faithfully,

S. W. Daw, F.R.C.S.,
Resident Surgical Officer Leeds General Infirmary.
General Infirmary, Leeds.
July 1.

[We have much pleasure in publishing the above letter, which is sensible and just, and on the whole we agree with its purport. Our protest was against the extremists who advocate methods of resistance upon lines which can have no other result but to injure the profession.—Ed., The Hospital.]

